# Receipt of Prescribed Controlled Substances by Adolescents and Young Adults Prior to Presenting for Opiate Dependence Treatment

**DOI:** 10.1155/2013/680705

**Published:** 2013-02-17

**Authors:** Steven C. Matson, Cathleen Bentley, Vicki Hughes Dughman, Andrea E. Bonny

**Affiliations:** ^1^Department of Pediatrics, The Ohio State University College of Medicine, Columbus, OH, USA; ^2^Division of Adolescent Medicine, Nationwide Children's Hospital, 700 Children's Drive, G353 Timken Hall, Columbus, OH 43205-2664, USA; ^3^Department of Clinical Services and Care Coordination, Nationwide Children's Hospital, Columbus, OH, USA

## Abstract

*Purpose*. The objective of this study was to document the number of controlled substance prescriptions filled by adolescents and young adult patients in the 2 years prior to presentation for opiate dependence treatment. *Methods*. Opiate-dependent youth (*N* = 125) presenting to our Medication-Assisted Treatment for Addiction program from January 1, 2008 to June 30, 2010 were identified via electronic medical record. Subjects were further classified based on their opiate use as dependent to heroin-only, prescription (Rx) opiate-only, or combined heroin + Rx opiate only. The Ohio Automated Rx Reporting System (OARRS) was used to identify each subject's controlled substance prescription history. Negative binomial regression was used to examine the relationships between patient characteristics and the total number of prescriptions filled. *Results*. Twenty-five percent of subjects had filled ≥6 prescriptions, and 15% had filled ≥11 prescriptions. The mean number of prescriptions filled was 5 (range: 0–59). Thirteen percent had filled ≥6 opiate/narcotic prescriptions, and 8% had filled ≥11 prescriptions. *Conclusions*. A subset of opiate-dependent youth had filled multiple opiate/narcotic prescriptions providing some evidence that physician-provided prescriptions may be a source of opiate abuse or diversion for a minority of opiate-dependent adolescents and young adults.

## 1. Introduction

The nonmedical use of Rx opiates and other controlled drugs by adolescents and young adults has surpassed all illicit drugs except marijuana [1]. A recent report found that, for youth, the peak risk in nonmedical use of prescription pain relievers occurred at the age of 16 years, not during the postsecondary school years as previously suspected [2]. According to the Monitoring the Future Study, the nonmedical use of several prescription medications by 12th graders in the United States is at its highest level in the past 15 years [3].

Concurrently, prescriptions by healthcare providers for controlled substances have also increased. A recent review of over 2.3 million visits by adolescents and young adults found that controlled medications were prescribed for an increasing proportion of adolescent (6.4 versus 11.2%) and young adult visits (8.3 versus 16.1%) between 1994 and 2007, respectively [4]. McCabe et al. found that the estimated prevalence of lifetime medical use of prescription opioids among US high school seniors was 17.6% while the estimated prevalence of lifetime nonmedical use was 12.9% [5]. McCabe has also demonstrated that the prevalence of medical misuse of controlled medication classes (pain, stimulant, sleeping, and anxiety) in adolescents was 18% and was higher among female versus male adolescents (20.8% versus 15.1%). Finally, medical misusers of controlled medications had a higher prevalence of all substance use and abuse behaviors than medical users who took their medications appropriately [6].

The precise role that legitimate prescriptions play in adolescent and young adult opiate dependence is not known. This study was specifically interested in examining the quantity of controlled medications prescribed by healthcare providers and filled by adolescents and young adults who later presented for opiate dependence treatment. The objective of this study was to document, utilizing a statewide database, the number of controlled substance prescriptions that adolescent and young adult patients filled in the two years prior to presentation for opiate dependence treatment.

## 2. Methods

### 2.1. Subjects

The study sample consisted of all adolescents and young adults, aged from 15 to 21 years, who presented to our Medication-Assisted Treatment for Addiction (MATA) clinic for outpatient treatment of opiate dependence from January 1, 2008 to June 30, 2010. The insurance mix of our MATA clinic is private insurance 35%, Medicaid 49%, and uninsured 16%. Although the General Adolescent Clinic which runs out of the same location sees a racially diverse patient population, patients who have presented to the MATA clinic to date have been predominantly Caucasian. Reasons for this are not entirely known but seem to reflect the demographics of the opiate-dependent population in our community. Data were extracted from the institution's electronic medical records database. There were no exclusionary criteria applied to the overall patient cohort except presentation to the MATA clinic for treatment of opiate dependence during the study period. The study protocol was reviewed and approved by the Institutional Review Board of the participating institution.

### 2.2. Data Collection

At each patient's initial visit to the MATA clinic a diagnosis of opiate dependence was confirmed using DSM IV-R criteria [7]. We then reviewed each subject's initial visit to the MATA clinic and obtained information on specific opiates used, gender, and age. Subjects were further classified based on their opiates use as (a) heroin-only dependent, (b) prescription (Rx) opiate-only dependent, or (c) combined heroin + Rx opiate dependent. 

As part of routine care in the MATA clinic, a patient's controlled substance prescription history for the two years prior to their intake visit was obtained utilizing the Ohio Automated Rx Reporting System (OARRS). The OARRS Prescription History Report provides information on the number and type of prescriptions for controlled substances filled by a patient during a specific time period. OARRS was established in 2006 as a tool to assist healthcare professionals in providing better treatment for patients with medical needs while quickly identifying drug seeking behaviors. Reports include all controlled substance prescriptions that were filled back until 2008 when complete reporting was available. Information includes the name of medications, the number of pills/capsules/films delivered, the prescribing physician, and the pharmacy that filled the prescription. An OARRS Prescription History Report can assist in assuring that a patient is getting the appropriate drug therapy and is taking medication as prescribed. 

We reviewed the OARRS Prescription History Report obtained on each subject at initial visit and collected the following information: (1) total number of prescriptions for controlled substances filled in the two years prior to presentation; (2) total number of pills for controlled substances obtained during the two years prior to presentation; and (3) specific type of controlled substances filled. Controlled substances filled by each subject were further categorized as belonging to one of the four following classes: (1) opiate/narcotic, (2) other analgesic (e.g., tramadol), (3) benzodiazepine, or (4) stimulant. We considered excluding stimulants from our data analyses since our clinical experience indicates that regular prescriptions for this class of medications are more common than for the other drug classes. However, we opted to keep stimulants in the analyses for the following reasons: (1) indications for receipt of controlled substance prescription were not known for any drug class, as such, speculation as to the indications of a prescription, stimulant or not, was not warranted; (2) a full picture of the controlled substances filled by our patients prior to presenting for opiate dependence treatment was desired; and (3) associations between stimulant prescriptions with gender and age were distinct from those seen for other drug classes and, hence, provided a meaningful contrast for interpretation of our study results. 

### 2.3. Data Analysis

Data analysis was conducted using SAS statistical software, version 6.12 (SAS Institute Inc., Cary, NC, USA). The main outcome measure was the total number of prescriptions for controlled substances filled in the two years prior to presentation to the MATA clinic. Secondary outcomes examined included the total number of pills for controlled substances filled in the two years prior to presentation and receipt of drugs from specific drug classes. 

Descriptive statistics included means, standard deviations, and range for continuous variables and percentages and counts for categorical variables. As appropriate for count data, negative binomial regression was used to examine the relationships between subject characteristics with the total number of prescriptions, total number of pills, and receipt of prescriptions from specific drug classes. The negative binomial dispersion parameter was estimated by maximum likelihood. Total number of prescriptions and total number of pills were highly correlated and as such demonstrated identical associations. Since the total number of prescriptions filled was our primary outcome of interest, final analyses focused on this outcome. Backward elimination negative binomial regression was used to build our final multivariate model. Eligible covariates for the multivariate model included gender, age, and type of opiate dependence.

## 3. Results

A total of 125 adolescents and young adults presented to the MATA clinic for treatment of opiate dependence from January 1, 2008 to June 30, 2010. The mean age of the study subjects was 18.5 ± 1.5 years. All subjects were Caucasian, and 59% were female. Forty-two percent of subjects were identified as heroin-only dependent, 37% as Rx opiate-only dependent, and 22% as combined heroin + Rx opiate dependent. Mean age and gender distribution were similar for all three opiate-dependent groups: heroin-only dependent (mean age 18.5 (±1.3); 60% female); Rx-opiate only dependent (mean age 18.5 (±1.7); 57% female); and combined heroin + Rx opiate dependent (mean age 18.5 (±1.4); 63% female). 

**Figure 1:**
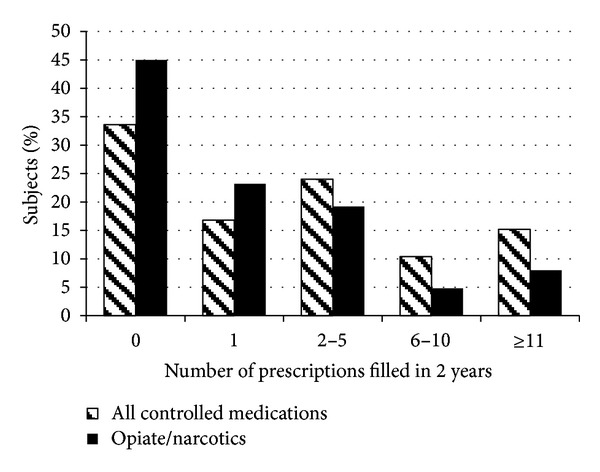
Distribution of total number of prescriptions for all controlled medications and opiate/narcotics filled in the 2 years prior to presentation for outpatient medication-assisted treatment for opioid dependence.

Two out of three patients had filled at least one prescription for controlled substances in the 2 years prior to intake, 25% had filled ≥6 prescriptions, and 15% had filled ≥11 prescriptions (Figure 1). The mean number of prescriptions filled was 5.3 (range: 0–59). The average number of pills or capsules dispensed was 194 pills (range: 0–2269). Examination of specific drug classes found that 54% of the subjects had filled a prescription for an opiate/narcotic, 13% had filled ≥6 opiate/narcotic prescriptions, and 8% had filled ≥11 prescriptions (Figure 1). Among opiate/narcotic prescriptions, specific medications prescribed to our study sample were as follows: hydrocodone/acetaminophen (43% of subjects); oxycodone (19% of subjects); codeine/acetaminophen (17% of subjects); codeine/cough formulation (3% of subjects); and meperidine (2% of subjects). Twenty-five percent of subjects filled a prescription for an analgesic, 18% for a benzodiazepine, and 13% for a stimulant.

In bivariate analyses, gender, age, and type of opiate dependence were all significantly associated with total number of prescriptions filled. Females filled on average 6.9 prescriptions versus 3.0 for males (*P* = 0.006). Subjects with Rx opiates-only dependence filled on average 8.7 prescriptions as compared to 3.3 for subjects with heroin-only dependence (*P* = 0.002) and 3.4 for subjects with heroin + Rx opiate dependence (*P* = 0.02). Increasing age was associated with a higher total number of prescriptions filled (maximum likelihood parameter estimate = 0.214; *P* = 0.02). In multivariate modeling, gender and type of opiate dependence remained independently associated with total number of prescriptions filled (Table 1).

Examination of prescriptions by specific drug classes showed some differences across gender, type of opiate dependence, and age (Table 2). Females were significantly more likely than males to have filled a prescription for benzodiazepines 24% versus 10%, respectively (*P* = 0.04). Although not statistically significant, filled opiate prescriptions were more common among subjects with Rx opiate-only dependence. Sixty-seven percent of patients with Rx opiate-only dependence had filled an opiate prescription, as compared to 46% of those with heroin-only dependence and 48% of those with combined heroin + Rx opiate dependence (*P* = 0.08). Mean age was significantly higher among patients who had filled an opiate (*P* = 0.004) or other analgesic (*P* = 0.001) prescription as compared to those who had not. In contrast, mean age was significantly lower for those who had filled a stimulant prescription as compared to those who had not (*P* = 0.001).

## 4. Discussion

This study found that the majority of adolescents and young adults presenting for opiate dependence treatment had filled at least one prescription for controlled medications in the preceding two years. Fifty-four percent had filled at least 1 prescription for opiate/narcotics. This percent is higher than that reported among the general adolescent and young adult population [4, 5]. Thirteen percent of our subjects had filled ≥6 prescriptions for opiate/narcotics in the two years prior to presentation. The high number of prescriptions filled by this subset of subjects suggests that physician-provided prescriptions may be a source of abuse or diversion for this subset of these opiate-dependent youths.

We have noted three distinct opiate-dependent subtypes in our treatment program: patients dependent on Rx opiates only, those dependent on heroin only, and those dependent on both. Among these opiate-dependent subtypes, we found variation in both the total number of filled prescriptions for controlled substances and the number of filled prescriptions from specific drug classes. Subjects with heroin-only dependence and those with both heroin + Rx opiate dependence showed a similar profile which was distinct from subjects with Rx opiates-only dependence. Those with Rx opiates-only dependence filled almost three times as many prescriptions for controlled substances as compared to the other two groups. Opiate prescriptions accounted for the largest difference in total number of prescriptions among these three groups.

Among our study subjects, females filled on average twice as many prescriptions for controlled substances as compared to males. Similarly, McCabe et al. found that females were more likely to have filled a prescription for a controlled medication as compared to male adolescents (56.4% versus 40.2%; *P* ≤ 0.001) [8]. Females are also significantly more likely than males to report nonmedical use of pain medication (22.2% versus 12.3%) [3]. The reasons behind these gender differences are not entirely clear. They could represent differences in patient behavior such as female adolescents asking for controlled substances with higher frequency or communicating symptoms in a distinct manner. In addition, they could represent provider differences in their approach to male and female patients.

Limitations of our current study include lack of racial diversity in our study sample and lack of information on receipt of controlled medications for nonmedical use from other sources such as peers. In addition, concurrent information on the number of prescriptions for controlled substances filled by adolescents without a diagnosis of substance abuse or dependence is not available. However, based on previously published data we expect that the past-year medical use of controlled medications by adolescents by medication class is as follows: 14.2% for pain medication, 2.2% for antianxiety medication, 1.6% for sleeping medication, and 3.5% for stimulant medication [6]. We also do not know the reasons why controlled medications were prescribed and filled by our subjects. Our clinical experience suggests that indications for opiates, other analgesics, and benzodiazepines in a general adolescent population are limited, and, except for the rare patient, multiple prescriptions for these controlled medications are not generally warranted. Regular prescriptions for stimulants, on the other hand, may in fact be indicated for some adolescent patients, and we considered removing stimulants from the current analyses. However, to get a complete picture of the controlled medications provided to our adolescent opiate-dependent patients, we opted to retain stimulants in our analyses. Interestingly, stimulants demonstrated a different relationship with both gender and age as compared to the other drug classes. Since stimulants were more prevalent in males and younger subjects, their effect on our final multivariate model would be to minimize the effect of female gender and increasing age on total number of prescriptions filled. Finally, information on concurrent psychiatric or substance abuse diagnoses was not documented on study subjects. This information will be included on future prospective data collection and subsequent reports.

Despite these limitations, our study has certain specific strengths. Very little is known about adolescent opiate-dependent patients. Prior research has primarily focused on Rx opiate use among a general adolescent population. Our study uniquely focuses on a population of adolescents with a diagnosis of opiate dependence. In addition, to our knowledge, our study is the first to utilize a state-automated prescription reporting database for documentation of prescriptions filled by an opiate-dependent population. We cannot know for certain what role filled prescriptions for controlled medications played in our study population. These prescriptions could have been for legitimate medical indications, nonmedical use (abuse), or diversion. However the small subset of subjects in our study who filled multiple prescriptions is concerning. It is possible that for this subset of subjects prescriptions were abused or diverted. McCabe et al. found that among youth receiving a controlled medication approximately 13.8% had ever sold, traded, loaned, or given away their medications [9]. The prevalence of substance use and abuse was significantly higher among prescribed users of controlled medications who had diverted their medications compared to their peers. Further research is needed to better understand the nature of use of prescribed medications among opiate-dependent youth.

Prescription surveillance systems like OARRS can be effectively utilized to better understand patterns of prescriptions of controlled substances among a dependent population and to identify “high volume” patients and providers. We found that a subset of our subjects had filled multiple prescriptions for controlled substances. Similarly, previous researchers found that 3% of physicians accounted for 62% of the narcotics prescribed in one study [10]. Since 1993 federal legislation has supported the formation of state-based prescription drug monitoring programs (PDMPs). To date, 42 states have operational PDMPs, and 6 have enacted legislation to develop programs. Both medical providers and patients would benefit from early identification of nonmedical prescription drug use and intervention prior to the problem escalating. Physicians should screen all adolescents or young adults for nonmedical use whenever prescribing a controlled medication including obtaining a controlled prescription report in states where available. Next steps should include identification of characteristics associated with transitioning from proper use of controlled medications to abuse and ultimate dependence. PDMPs like OARRS could possibly be utilized in the future to identify points at which individuals transition from proper use to abuse.

## 5. Implications and Contributions

The nonmedical use of prescription opiates by adolescents and young adults has surpassed all illicit drugs except marijuana. The exact contribution of prescribed medications to the development of opiate dependence is not clear. Our findings provide some evidence that physician-provided prescriptions may be a source of abuse or diversion in a subset of opiate-dependent youth.

## Figures and Tables

**Table 1:** Multivariate^a^ negative binomial regression predicting the total number of prescriptions for controlled substances filled in the 2 years prior to presenting for outpatient medication-assisted treatment for opiate dependence.

Parameter	Parameter estimate^b^	95% confidence interval		*P* value
Intercept	1.638	1.053	2.224	<0.0001
Type of opiate dependence				
Heroin only	−0.900	−1.522	−0.278	0.005
Heroin + Rx opiate	−0.866	−1.613	−0.119	0.023
Rx opiate only	Reference	—	—	
Gender				
Female	0.711	0.143	1.278	0.014
Male	Reference	—	—	
Dispersion	2.204	1.627	2.986	

^a^Eligible covariates included age, gender, and type of opiate dependence.

^b^The negative binominal dispersion parameter was estimated by maximum likelihood.

**Table 2:** Likelihood of filling prescriptions for specific drug classes by gender, type of opiate dependence, and age.

Characteristic	Opiate	Other analgesic	Benzodiazepine	Stimulant
Gender:				
% of females who filled each drug class	58.1	29.7	24.3^a^	10.8
% of males who filled each drug class	49.0	17.6	9.8^a^	15.7
Type of opiate dependence:				
% of Rx opiate-only dependents who filled each drug class	67.4	21.7	23.9	17.4
% of heroin + Rx opiate dependents who filled each drug class	48.2	37.0	18.5	3.7
% of heroin-only dependents who filled each drug class	46.2	21.2	13.5	13.5
Mean age by receipt of specific drug class:				
Yes	18.8^b^	19.3^b^	18.9	17.2^b^
No	18.1^b^	18.2^b^	18.4	18.7^b^

^a^Comparison significant at *P* < 0.05.

^b^Comparison significant at *P* < 0.01.

## Abbreviations

MATA: Medication-assisted treatment for addiction
OARRS: The Ohio automated Rx reporting system
Rx: Prescription
PDMP: Prescription drug monitoring program.

## References

[1] Substance Abuse and Mental Health Services Administration *Results From the 2010 National Survey on Drug Use and Health: Summary of National Findings*.

[2] Meier EA, Troost JP, Anthony JC (2012). Extramedical use of prescription pain relievers by youth aged 12 to 21 years in the United States: national estimates by age and by year. *Archives of Pediatrics & Adolescent Medicine*.

[3] Johnston L, O'Malley P, Bachman J *Monitoring the Future National Results on Adolescent Drug Use: Overview of Key Findings*.

[4] Fortuna RJ, Robbins BW, Caiola E, Joynt M, Halterman JS (2010). Prescribing of controlled medications to adolescents and young adults in the United States. *Pediatrics*.

[5] McCabe SE, West BT, Teter CJ, Boyd CJ (2012). Medical and nonmedical use of prescription opioids among high school seniors in the United States. *Archives of Pediatrics & Adolescent Medicine*.

[6] McCabe SE, West BT, Cranford JA (2011). Medical misuse of controlled medications among adolescents. *Archives of Pediatrics and Adolescent Medicine*.

[7] American Psychiatric Association (2000). *Diagnostic and Statistical Manual of Mental Disorders*.

[8] McCabe SE, Boyd CJ, Young A (2007). Medical and nonmedical use of prescription drugs among secondary school students. *Journal of Adolescent Health*.

[9] McCabe SE, West BT, Teter CJ (2011). Characteristics associated with the diversion of controlled medications among adolescents. *Drug and Alcohol Dependence*.

[10] Swedlow A, Ireland J, Johnson G (2011). *PreScribing Patterns of Schedule II Opioids in California Worker’s Compensation*.

